# Agro-Morphological, Microanatomical and Molecular Cytogenetic Characterization of the Medicinal Plant *Chelidonium majus* L.

**DOI:** 10.3390/plants9101396

**Published:** 2020-10-20

**Authors:** Tatiana E. Samatadze, Olga Y. Yurkevich, Firdaus M. Hazieva, Elena A. Konyaeva, Alexander I. Morozov, Svyatoslav A. Zoshchuk, Alexandra V. Amosova, Olga V. Muravenko

**Affiliations:** 1 Engelhardt Institute of Molecular Biology, Russian Academy of Sciences, 32 Vavilov St, 119991 Moscow, Russia; tsamatadze@gmail.com (T.E.S.); olikys@gmail.com (O.Y.Y.); slavazo@mail.ru (S.A.Z.); olgmur1@yandex.ru (O.V.M.); 2 All-Russian Scientific Research Institute of Medicinal and Aromatic Plants, 7 Green St, 117216 Moscow, Russia; vilar.6@yandex.ru (F.M.H.); lencon64@mail.ru (E.A.K.); vilaragro@mail.ru (A.I.M.)

**Keywords:** *Chelidonium majus* L. subsp. *majus*, bio-morphology, phytotomy of leaves and flowers, meiosis, karyotype, fluorescence in situ hybridization (FISH)

## Abstract

*Chelidonium majus* L. is a medicinal plant well-known as a valuable source of isoquinoline alkaloids, which has a variety of pharmacological properties including anti-viral and anti-bacterial effects. However, considerable intraspecific bio-morphological variability in *C. majus* complicates raw material identification and verification. For the first time, we have brought into cultivation five populations of *C. majus* subsp. *majus* originated from different regions, and performed their agro-morphological, microanatomical and molecular cytogenetic characterization. All examined populations produced high seed (18.6–19.9 kg/ha) and raw material (0.84–1.08 t/ha) yields; total alkaloid contents were within 0.30–0.38%. Nevertheless, significant differences in plant morphology and yield-contributing traits were observed. The performed microanatomical analysis of leaves and flowers in double- and normal-flowered plants revealed micro-diagnostic features (including tissue topography, types of stomata, laticifers, structure of leaf mesophyll, hairs, sepals and petals) important for identification of *C. majus* raw materials. The analysis of chromosome morphology, DAPI-banding patterns, FISH mapping of 45S and 5S rDNA and also chromosome behavior in meiosis allowed us to identify for the first time all chromosomes in karyotypes and confirm relative genotype stability of the studied plants. Our findings indicate that the examined *C. majus* populations can be used in further breeding programs.

## 1. Introduction

Herbal medicine is considered to be an effective alternative to chemical medicine. Wild and introduced medicinal plants are used as a source of raw materials for obtaining a lot of effective remedies. Currently, about forty percent of medicines comprise herbal active ingredients, and the demand for medicinal herbs continues to increase [1,2].

Greater celandine, *Chelidonium majus* L. (*Papaveraceae*), is a valuable medicinal plant which is widely distributed in Europe and Asia and also introduced in Northern America. *C. majus* is a plant of great interest for its wide use in folk medicine and also in official phytotherapy. This species is known to synthesize a broad range of secondary metabolites which provide its therapeutic properties [3]. The most common group of these secondary metabolites are isoquinoline alkaloids (1–2% in the herb and 2–4% in the roots), including chelidonine, berberine, sanguinarine, coptisine, chelerythrine and protopine [4]. Besides, *C. majus* contains flavonoids, saponins, vitamins (e.g., vitamin A and C), mineral elements, sterols, and acids and their derivatives [5]. Pharmacological properties ascribed to *C. majus* include anti-bacterial [6], anti-inflammatory [7], anti-viral [8], anti-fungal [9], anti-protozoal and radioprotective [10], hepatoprotective [11], natriuretic and antidiuretic and also anti-alzheimer effects [12]. In recent years, an anti-cancer potential of various *C. majus* alkaloids including chelidonine, sanguinarine, berberine, chelerythrine and coptisine, has been demonstrated, and the interest in Greater celandine as a natural crude drug has increased significantly [4,13,14,15,16]. In particular, the methanol extract of *C. majus* and the coptisine alkaloid showed strong cytotoxicity against human colon carcinoma [17]. In addition, *C. majus* is used to produce a semi-synthetic drug NSC-631570 called Ukrain that was shown to exhibit selective toxicity towards malignant cells and promoted as a potential anti-cancer agent [18,19]. Recently, the molecular mechanisms of alkaloid synthesis in *C. majus* have been investigated based on de novo transcriptome sequencing of leaf and root tissues using Illumina technology [20].

Until 1982, the genus *Chelidonium* L. was considered to be monotypic with *C. majus* L. as the only species. Later, based on studies of the species cytotaxonomy and distribution areas, this genus was divided into two species: *C. majus* L. (2n = 12) native to Europe, northern Africa and western Asia, and *C. asiaticum* (Hara) Krahulc. (2n = 10) native to eastern Asia [21,22,23]. Alongside the differences in chromosome numbers and distribution areas, these species are slightly different morphologically. *C. asiaticum* is hairier, with narrower and sharper leaf lobes compared to *C. majus*. Besides, within *C. majus*, two subspecies: *C. majus* L. subsp. *majus* (distributed in Europe) with more laciniate lobes of leaves and *C. majus* L. subsp. *grandiflorum* (DC.) Printz (native to South Siberia and China) were distinguished [21]. Molecular phylogenetic studies of *Papaveroideae* based on DNA sequences of chloroplast rbcL and matK and also nuclear ITS regions indicated that the members of *Chelidonieae* formed a strongly supported monophyletic clade in the current tree [24]. Recently, the whole chloroplast genome of *C. majus* has been sequenced, and these data could be useful for further study of the phylogenetic relationships within the family *Papaveraceae* [25].

Due to a wide geographical distribution of greater celandine, considerable variability in morphological, biochemical and other characters was observed [26,27,28]. Specifically, in different *Chelidonium* samples, the species identification and therefore, validation of raw materials, are complicated by a significant variability of morphological characters, which depends on edaphic, climatic and other environmental conditions. For adequate investigation of intra- and inter-population variability of greater celandine, a complex of diagnostic characters for assessments of raw materials should be used [29].

Moreover, currently available karyological data on *Chelidonium* are based on simple monochrome staining of chromosomes, and for *C. majus*, only basic number of chromosomes and their sizes (ranged from 2 to 7 µm in different reports) have been determined to date [21,30,31]. The application of modern molecular cytogenetic techniques will make it possible to obtain the additional information about structural organization of the *C. majus* genome that could be useful for breeding efforts focused on developing new valuable cultivars.

In the present study, for the first time, we have brought into cultivation five samples of *C. majus* subsp. *majus* originated from different regions, and performed their agro-morphological, microanatomical and molecular cytogenetic characterization. This approach aimed to estimate the intraspecific variability essential for potential breeding programs and also reveal new diagnostic characters of *C. majus* raw materials.

## 2. Results

### 2.1. Agro-Morphological Peculiarities

The growing period of the *C. majus* plants lasted for 173–178 days (from April to September). Plants of the studied populations were highly resistant to the local winter conditions (approximately ranged from 3 to −20 °C). The number of overwintered plants in the newly introduced samples was comparable to the control (90–96%).

**Figure 1:**
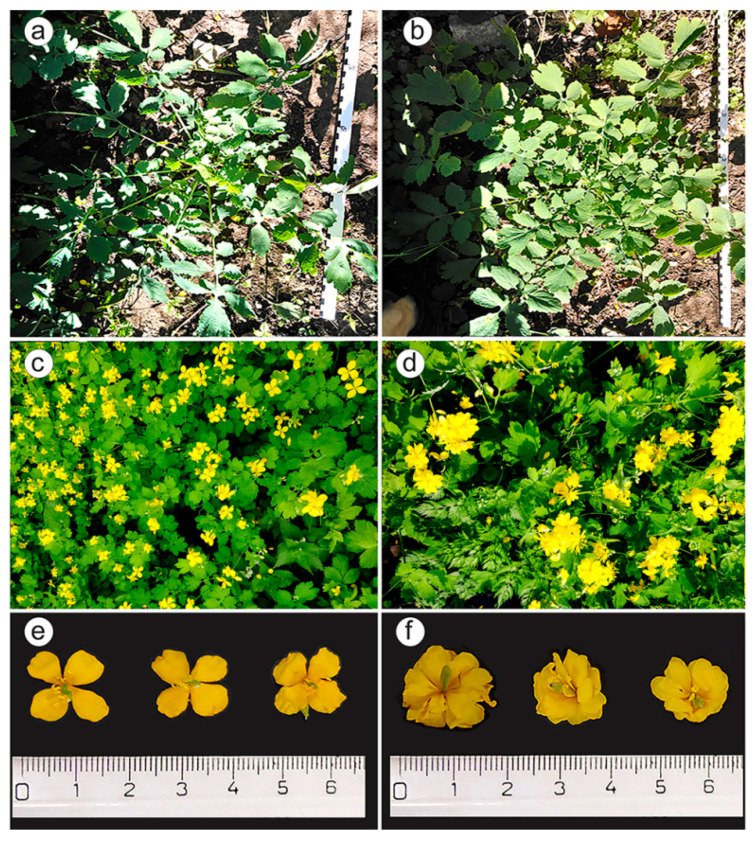
*Chelidonium majus* plants and inflorescences in the control normal-flowered (K 171-1) and double-flowered (K 211) populations. Plants in the rosette growth stage: (**a**) K 171-1, (**b**) K 211; flowering plants: (**c**) K 171-1, (**d**) K 211; inflorescences: (**e**) K 171-1, (**f**) K 211.

The examined *C. majus* plants were up to 80 cm high with branched and sparsely pubescent stems and green alternately placed leaves (Figure 1).

The basal (rosette) leaves had long petioles and pinnatodentate leaflets with 5–7 lobed segments (Figure 1a,b). The apical leaves had short petioles and leaflets with 3–5 lobed segments (Figure 1c,d). The plants produced umbellate inflorescences with 2–6 flowers (Figure 1c,d). Normal-flowered plants had four bright yellow petals and two whitish sepals (Figure 1e). Plants of the double-flowered sample (K 211) had 24.6 ± 1.56 petals in a flower and differed in flower morphology compared to the normal-flowered samples (Figure 1e,f; Table 1). Fruits were multi-seeded, pod-shaped and elongated (4–4.5 cm long) (Table 1). The seeds are shiny, ovate and dark brown. All parts of the plant except flowers contain yellow-orange latex.

Plants in the studied *C. majus* populations were not significantly different in several morphological features, including number of flower spikes, number of flowers per plant, flower diameter, number of seeds per fruit and length of fruits (Table 1).

**Figure 2:**
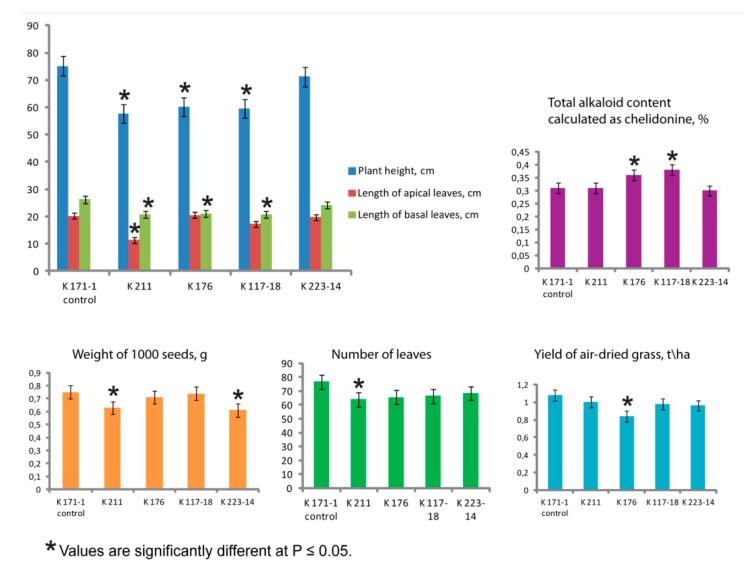
Bio-morphological traits of plants in the studied *Chelidonium majus* populations. Plant height (cm), length of apical and basal leaves (cm), total alkaloid content calculated as chelidonine (%), weight of 1000 seeds (g), number of leaves and yield of air-dried grass (t/ha) (the vertical axis) in the studied samples (the horizontal axis).

At the same time, the examined plant populations differed in the mean value of plant height, which was higher in the control K 171-1 and K 223-14 samples compared to the other samples (K 176, K 117-18 and K 211). Plants of both K 171-1 and K 223-14 samples also had the increased length of basal and apical leaves (Figure 2).

Plants of the double-flowered sample (K 211) displayed decreased length of apical leaves, number of leaves and number of flower spikes compared to plants of the control (K 171-1) sample having the same origin (Moscow region) as well as the other *C. majus* samples from other regions (Figure 2).

The mean values of yields of air-dried grass varied from 0.84 to 1.08 t/ha, and the control sample (K 171-1) had the highest yield (1.08 ± 0.11 t/ha). Also, the plants of this sample produced the most leaves (76.50 ± 4.75) (Figure 2). Values of seed yields were rather high in all populations (Table 2). At the same time, plants of the studied *C. majus* samples differed in the value of weight of 1000 seeds which was higher in samples K 171-1 (control), K 176 and K 117-18 compared to K 211 and K 223-14 (Figure 2).

Besides, plants of the examined *C. majus* samples differed in total alkaloid contents which ranged from 0.30% to 0.38%. The samples K 171-18 and K 176 had higher total amount of alkaloids (0.38 ± 0.02 and 0.36 ± 0.01) compared to the control (0.31 ± 0.01) (Figure 2).

### 2.2. Microanatomical Analysis of Leaves and Flowers

**Figure 3:**
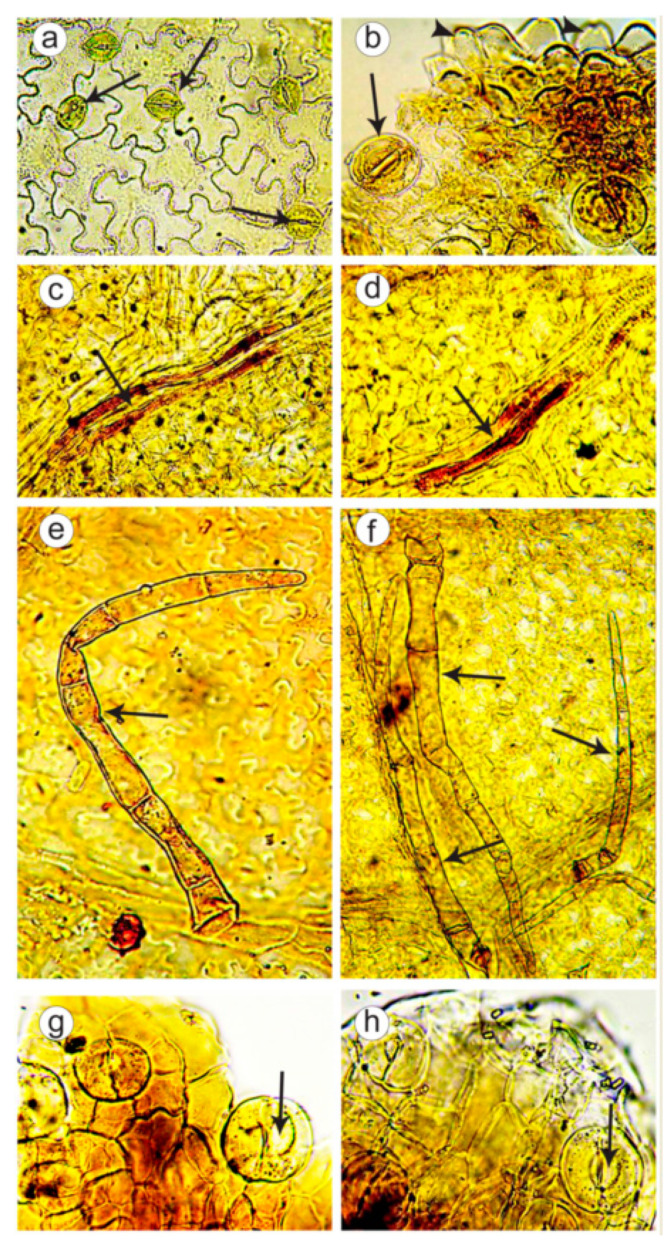
Micro-morphology of the epidermis of leaves and flowers in plants of the control normal-flowered (K 171-1) and double-flowered (K 211) populations of *Chelidonium majus*. Cells of the lower epidermis of a leaf: (**a**) K 211, cell with stomata (arrows) (magnification ×200), (**b**) K 171-1, cell with stomata (arrows) and crenate teeth with nipple-shaped cells (arrow heads) (×400). Lactiferous cells (arrows) observed along a leaf vein (×400): (**c**) K 211, (**d**) K 171-1. Simple long pluricellular hairs (arrows) on the leaf epidermis: (**e**) K 211, (**f**) K 171-1 (×200). Top of a sepal with water stomata (arrows) (×400): (**g**) K 211, (**h**) K 171-1.

A microscopic analysis of leaves and flowers was performed in plants of double-flowered (K 211) and control normal-flowered (K 171-1) samples. In both *C. majus* samples, cells of the leaf epidermis had flexuous configuration, especially on the lower part of a leaf. In cells of the lower epidermis, the anomocytic stomata, surrounded by 4–7 (more rarely, 3–8) epidermal cells, were observed. In some places, radiate folds of the cuticle were revealed (Figure 3a,b). On tops of the crenate teeth, where leaf veins converged, epidermal cells were nipple-shaped and also large water stomata were found (Figure 3b). Spongy cell tissue formed intercellular space (aerenchyma tissue). Leaf veins had lactiferous cells with yellowish-brown granular contents (Figure 3c,d). On the lower leaf epidermis, along the veins, simple long pluricellular (up to 20 cells) thin-walled hairs were observed, which were often overwound, crumpled or had few collapsed cells (Figure 3e,f). In plants of the double-flowered population, fewer hairs were observed compared to the control. 

In flowers of both double-flowered and control samples, sepal epidermis had longwise elongated cells with straight or slightly flexuous walls. Along the veins subjacent the sepal, lactiferous cells were revealed. In examining of a petal, longwise elongated epidermal cells with straight or slightly flexuous walls and yellowish-brown contents were observed. Outside the petal, large anomocytic stomata were detected. Besides, water stomata were found on top of the sepal (Figure 3g,h).

### 2.3. Chromosome Structure

**Figure 4:**
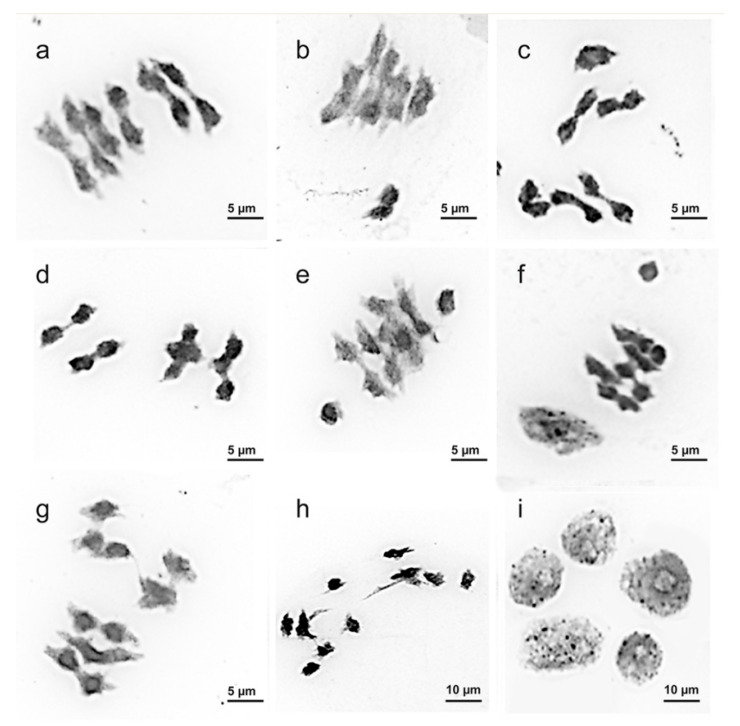
Chromosome behavior during meiosis observed in the studied *Chelidonium majus* plants: (**a**) M-I, 6^II^, (**b**) M-I, several chromosomes are localized outside the metaphase plate, (**c**) M-I, 4^II^ + 1^IV^, (**d**) M-I, 2^II^ + 2^IV^, (**e**) M-I, 5^II^ + 2^I^, (**f**) M-I, 5^II^ + 1^I^, (**g**) A-I, chromosome lagging, (**h**) A-I, chromosome bridges and chromosome lagging, (**i**) pentad.

Analysis of meiosis in maternal pollen cells of the examined *C. majus* samples indicated 98.41% regular meiotic chromosome behavior with normal chromosome disjunction and six bivalents (6^II^) at metaphase I (M-I) (Figure 4a). Besides, few chromosome associations (trivalents and quadrivalents) and also univalents lying outside the metaphase plate (on the side of the plate or at the cellular poles) were revealed (Figure 4b–f). At anaphase I (A-I) and anaphase II (A-II), 97.83% of cells of the studied samples had normal chromosome disjunction (6:6). However, several abnormalities (chromosome lagging, bridges, fragments, chaotic disjunction, unequal distribution of genetic material, etc.) were also observed (Figure 4g,h). At the tetrad stage, normal tetrads (97.76%) as well as few polyads (pentads and hexads) and misoriented tetrads were detected (Figure 4i). The cumulative percentage of microsporocytes with meiotic abnormalities observed in the studied *C. majus* samples was negligible (2.17%).

**Figure 5:**
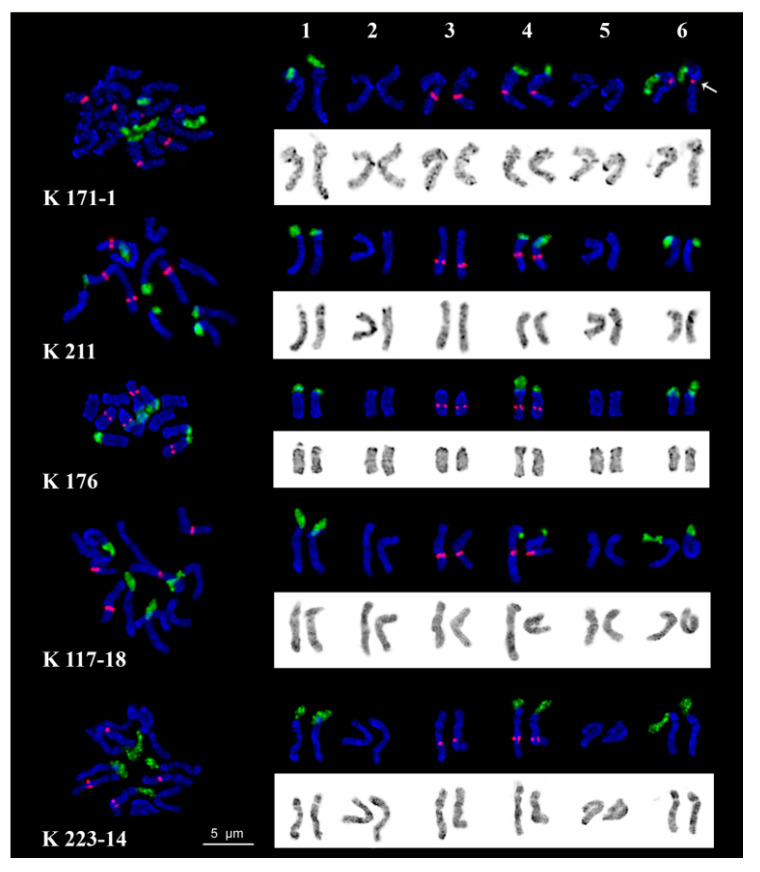
Karyotypes of the studied *Chelidonium majus* plants. Metaphase spreads and karyograms after FISH with 45S (green) and 5S (red) rDNA. DAPI banding patterns are shown as inverted images. Arrow points to the minor polymorphic 5S rDNA loci. Scale bar = 5 μm.

Karyotypes of the studied *C. majus* plants contained six metacentric and submetacentric chromosome pairs (2n = 2x = 12) including three satellite chromosomes pairs. Chromosome lengths ranged from 4.5 to 6.5 µm. The satellite chromosomes had similar morphology with the secondary constriction located in the pericentromeric region of the short arms. Large DAPI-bands were observed in the pericentromeric and terminal regions, while small and middle-sized bands were located in the intercalary regions of chromosomes. DAPI-staining produced a characteristic pattern on each *C. majus* chromosome pair, though individual variations in DAPI-banding patterns were revealed (Figure 5).

In all examined *C. majus* karyotypes, clusters of 45S rDNA were revealed in the secondary constriction regions of satellite chromosome pairs 1, 4 and 6, and 5S rDNA sites were detected in the median regions of the long arms of chromosome pairs 3 and in the pericentromeric regions of the long arms of chromosome pair 4. Also, in some individuals, size heteromorphism of 45S rDNA clusters between homologous chromosomes was observed (shown, in particular, in chromosome pair 4 in the K 176 karyotype). Besides, in karyotypes of sample K 171-1, a minor polymorphic 5S rDNA locus was revealed in the pericentromeric regions of satellite chromosome pair 6 (Figure 5). Based on chromosome morphology, DAPI-banding patterns and distribution of 45S and 5S rDNA loci, all chromosome pairs in karyotypes were identified and chromosome idiograms of plants from each studied *C. majus* populations were constructed (Figure 5).

## 3. Discussion

Bio-morphological characterization is one of the most universal and available approaches to estimate the plant intraspecific diversity [32]. Every plant genotype has definite traits including plant height, length and width of leaves, size and shape of flowers and fruits, trichome morphology, and color of leaves, seeds, flowers and fruits, which are used for identification of species, cultivars or hybrids, as well as verification of their homogeneity and stability [33]. In this respect, plants in the studied *C. majus* populations were not significantly different in several morphological features, including number of flower spikes, number of flowers per plant, flower diameter, number of seeds per fruit and length of fruits. At the same time, the studied populations differed in lengths of basal and apical leaves, which were longer in plants of the control K 171-1 and K 223-14 samples. Besides, the examined plants had longer fruits (4–4.5 cm) compared to the earlier described data (3 cm) [3]. The detected morphological variations were probably due to considerable intra- and inter-population variability in bio-morphological characters which could be related to genome plasticity facilitating wide geographical distribution of this species [26,27,28].

We also found that the value of plant height in the studied *C. majus* samples was less (up to 80 cm high) if compared with the data reported earlier (up to 100 cm high) [3]. Moreover, the examined populations were significantly different in plant height, which was higher in samples K 171-1 and K 223-14 compared to the other three populations. An optimal value of plant height is considered to be an important agronomic trait which contributes to a high yield as short plants are more resistant to lodging than tall ones. Currently, breeding efforts for many medicinal plants are aimed to growth limitation of lateral meristems in joints of a plant stem, and in this connection, short-stemmed populations can be useful for the further breeding process [34].

In the present study, plants of double-flowered morphotypes were revealed within the normal-flowered *C. majus* population (K 171-1) and then cultivated as a separate population (K 211). It was previously shown that double-flowered plants could appear spontaneously within normal-flowered populations due to the impact of natural or artificial mutagenic factors [35,36]. According to our observations, double-flowered *C. majus* plants were short-stemmed, had high fertility rate and produced viable seeds and stable yields. These characteristics indicated that they could be used as potential genetic resources in breeding programs with a view to developing new ornamental cultivars.

Several characters, including length of growing seasons, winter hardness and seed productivity, indicate how the recently introduced plants have adapted to the regional environments and growing conditions [37]. In the present study, the vegetation period of the examined *C. majus* plants lasted from April to September (173–178 days long) and did not exceed the average length of a growing season for agricultural crops (200–220 days) observed in the local (Moscow) region [38]. Besides, the studied *C. majus* plants were highly resistant to winter conditions (90–96% of overwintered plants). Agronomic characterization of the studied *C. majus* populations indicated that they were rather high yielding. All examined *C. majus* populations demonstrated high yield potentials for producing both seeds (18.6–19.9 kg/ha; 48.2–51.2 seeds per fruit) and raw materials (0.84–1.08 t/ha).

Isoquinoline alkaloids are considered to be pharmacologically relevant substances of *C. majus* [39]. It is known that alkaloid contents in different individual plants (and even in parts of plants) are not stable [40]. These differences can depend on genotype (chemotype), plant age, plant part, developmental stage or environmental conditions [41]. The examined *C. majus* samples were different in total alkaloid contents which ranged from 0.30% to 0.38%. Two samples (K 171-18 and K 176) had higher contents of alkaloids (0.38% ± 0.02% and 0.36% ± 0.01%, respectively) compared to the control (0.31% ± 0.01%). Nevertheless, according to the requirements of the Pharmacopoeia of RF on *C. majus* [42], the total contents of alkaloids calculated as chelidonine should not be less than 0.2%. All studied *C. majus* populations complied with these requirements, which might be indicative of the high quality of these raw materials.

The identification and estimation of quality of *C. majus* raw materials are especially important for its medicinal use. The microscopic authentication is considered to be one of the most important methods for the standardization and quality control of drug raw materials. Particularly, a microscopic analysis is applied for accurate identification and/or determination of a level of contamination in a plant sample [43]. Besides, structural features of the epidermis of leaves and flowers are often used as the verification standard in harvesting of the aboveground phytomass [44,45]. In this regard, the important diagnostic characters for identification of plant raw materials include shapes of the epidermal cells, structure of leaf mesophyll, sepals and petals, presence and types of stomata, presence and structure of hairs and glands, presence of water stomata, crystal inclusion, secretory cells and laticifers [45]. Isoquinoline alkaloids are the components of latex which is produced in all parts of *C. majus* plants, except flowers. Latex is stored in the special secretory cells called laticifers. According to Pharmacopoeial monographs, presence of articulate laticifers with yellowish content is used as an authentication microscopic mark in powdered herb [40,42,46].

In the present study, we performed for the first time a microscopic examination of the epidermis of leaves and flowers in double-flowered (K 211) and control normal-flowered (K 171-1) *C. majus* plants. We revealed a number of common features in their internal structure, which characterize this medicinal plant and could be used for raw material identification. Particularly, these common micro-diagnostic features of *C. majus* plants included tissue topography (curvilinear thin-walled cells of epidermis), anomocytic types of stomata, presence of water stomata and laticifers, peculiarities in structure of leaf mesophyll, sepals and petals, and also types and structure of hairs on the lower leaf epidermis. At the same time, in plants of the double-flowered population, we observed fewer specific hairs on the lower leaf epidermis if compared with the control. These differences could also be a manifestation of the intraspecific bio-morphological variability described earlier [26,27,28].

The analysis of chromosome behavior during meiosis is an effective tool for detection of different chromosome abnormalities at gametogenesis, which can further result in pollen sterility and decrease seed production. This approach makes it possible to identify the most stable plant genotypes which can be used as genetic resources in further breeding programs [47].

In microsporocytes of the examined *C. majus* plants, normal meiotic segregation patterns (bivalents, 6:6) were observed, though few univalents, trivalents and quadrivalents were also detected. As the result of such disorders, cells with different chromosome abnormalities (chromosome lagging, bridges, fragments, chaotic disjunction, unequal distribution of genetic material, etc.) could appear at the following stages of meiosis. Such chromosome aberrations can be related to the spindle assembly defects and result in formation of gametes with unbalanced chromosome number [48]. At the tetrad stage, normal tetrads were observed, though we also revealed several pentads and misoriented tetrads which were probably associated with defects in achromatic spindle orientation [49]. The above mentioned meiotic abnormalities were detected in many plant species [50,51]. They are considered to be related to some genetic [52,53] and/or environmental [54,55,56] factors. Such chromosomal disorders can result in sterility of gametes and low percentage of pollen viability [57]. In the studied *C. majus* populations, however, the percent of microsporocytes with meiotic abnormalities was insignificant, indicating the relative genotype stability of the examined plants.

Currently available data on the *C. majus* karyotype show that it contains twelve metacentric and submetacentric chromosomes, including one pair of satellite (SAT) chromosomes [21,30,31]. However, these reports provided different information about the sizes of its chromosomes: 2–3 µm [31] and 5–7 µm [30]. This can be related to differences in methods used for preparation of metaphase chromosome spreads from root meristem, which resulted in different levels of chromosome condensation. At the same time, the comprehensive examination of *C. majus* chromosomes with the use of molecular markers has not previously been conducted, though data on its transcriptome sequencing have already been obtained [20]. In the present study, for the first time, we have studied the karyotype of *C. majus* subsp. *majus* with the use of FISH with 45S and 5S rDNA. Besides, we analyzed chromosome DAPI-banding patterns displaying (after FISH) AT-rich heterochromatin stains in chromosomes of vascular plants. This approach is considered to be a useful tool to reveal chromosome structural variations among populations [58]. Eukaryotic 45S and 5S rRNA genes are clustered in tandem repeats which can localize in one or several chromosome pairs. Due to the fact that rRNA genes are highly conserved, rDNA clusters are easily mapped by FISH on chromosomes in different species and widely used as chromosomal markers in plant karyological studies [59].

At the same time, detailed analysis of karyotypes of plant species having small-sized chromosomes needs special approaches. In view of this, during the stage of chromosome preparation from root meristem, we applied nonspecific DNA intercalator 9-AMA, which was shown to inhibit the chromosome condensation process and facilitate the accumulation of metaphase cells with long chromosomes [60]. As a result, the obtained *C. majus* karyotypes contained chromosomes ranging from 4.5 to 6.5 µm (depending on the chromosome condensation level). Chromosome DAPI-banding patterns were used as an additional parameter for the identification of the homologous chromosomes. Visual analysis showed that these patterns were specific to each chromosome pair though low intra- and inter-individual variability in presence and sizes (intensity) of DAPI-bands was also observed. Polymorphism on chromosome DAPI-banding patterns (AT-rich heterochromatin chromosome regions) is considered to be related to structural peculiarities of chromosomes [61].

In all studied *C. majus* karyotypes, the FISH procedure revealed 45S rDNA loci in three chromosome pairs, 1, 4 and 6. The size heteromorphism of 45S rDNA clusters observed between homologous chromosomes of some individuals can be related to different activity of NORs in these chromosomes, which was also detected in other species of plants and animals [36,62]. Major 5S rDNA clusters were revealed on two chromosome pairs, 3 and 4. Besides, in karyotypes of plant population K 171-1, a minor polymorphic 5S rDNA locus was revealed in the pericentromeric region of SAT chromosome pair 6. It was demonstrated earlier that the number of 5S rRNA gene copies could vary in different plant ecotypes, and those changes could be related to plant genome plasticity [63,64]. At the same time, considerable chromosome abnormalities including aneuploidy or chromosome rearrangements were not detected in plants of the examined *C. majus* populations, which is indicative of a relative stability of their karyotypes. The constructed chromosome karyograms can be useful for karyotype comparisons in different *C. majus* populations as well as for further comparative karyological studies of taxa within the genus *Chelidonium*, important for verification of their phylogenetic relationships.

## 4. Materials and Methods 

### 4.1. Plant Material

In the present study, we used seeds of five samples of *C. majus* subsp. *majus* originated from different regions (Table 2) and obtained from the seed collection of All-Russian Scientific Research Institute of Medicinal and Aromatic Plants, Moscow region, Russia. These plant samples were cultivated in the fields of the Botanic Garden of the All-Russian Institute of Medicinal and Aromatic Plants during three successive experimental seasons (summer 2017–summer 2019). The indigenous *C. majus* population K 171-1 was used as a control. In 2016, double-flowered *C. majus* plants spontaneously arose within the normal-flowered (control) population, and seeds of those (double-flowered) plants were collected. Then, the double-flowered plant population was cultivated as a separate sample (K 211).

At late April 2017, seeds of samples K 171-1, K 211, K 176, K 117-18 and K 223-14 were planted on the open-field trial plots having a randomized block design. Total and accounting areas of the plots were 8.4 and 3.6 m^2^ respectively, with four-fold replication.

The soil cover of the trial plots was represented by sod-podzolic (moderately podsolized) silt loams (80–100 cm) underlain by moraine deposits. The plough-layer (22–23 cm) was brownish-grey and had crumbly structure and sandy loam soil composition. The content of healthy water-stable aggregates (>0.5 mm) was 40–50%. The soils of the trial plots had the following nutritional characteristics: humus content—2.1%, pH 5.5, the content of mobile phosphorus (P_2_O_5_)—52 mg/kg and exchangeable potassium (K_2_O)—87 mg/kg. In primary cultivation, fertilizer treatment with N_30_P_30_K_30_ was performed according to the standard techniques described earlier [65].

### 4.2. Bio-Morphological Characterization

Plant vegetative parameters including: plant height, number of flower spikes, number of flowers per plant, flower diameter, number of petals in a flower, number of seeds per fruit and length of fruits, length of apical and basal leaves, number of leaves, yield of grass and seeds were determined at four phenological growth stages: leaf development, flower-bud formation, flowering and fruit maturity according to the standard techniques [66]. At least 50 plants were analyzed from each population. In each vegetation period, the yield of grass (raw material) was cut using one-cut harvest method at the stage of flowering. The yield of seeds was harvest at the stage of fruit maturity [67]. The statistical data analysis was performed using standard functions of Microsoft Excel 2013. In April, plant resistance to winter low temperatures was determined as a number of live plants at the beginning of growth of basal rosette leaves in spring.

### 4.3. Chemical Composition of the Grass

Plant raw material (herbs of *C. majus* cut at the stage of plant flower maturity) was ground into a powder. Total alkaloid content in plant raw material was determined according to the State Pharmacopoeia of the Russian Federation with the use of Agilent Technology 6890N gas chromatograph (Agilent Technology, Santa Clara, CA, USA) [42].

### 4.4. Chromosome Spread Preparation

Mitotic chromosome spreads were prepared from plant root meristem with the use of DNA intercalator 9-aminoacridine (9-AMA) according to the technique developed previously [60]. Plant seeds were germinated on the moist filter paper in Petri dishes at room temperature (RT). Root tips (of 0.5–1 cm long) were excised and stored for 16–20 h in ice-cold water with 1 µg/mL of 9-aminoacridine (9-AMA) (Sigma, St. Louis, MO, USA) to inhibit chromosome condensation process and accumulate mitotic divisions. Then, the root tips were fixed in Carnoy’s fixative (ethanol:acetic acid (3:1)) for 48 h at 4 °C. Before squashing, the roots were transferred into 1% acetocarmine solution in 45 % acetic acid for 15 min. The cover slips were removed after freezing in liquid nitrogen. The slides were dehydrated in 96 % ethanol and stored at −20 °C until use.

For meiotic chromosome preparation, young flower buds (2–5 mm long) were fixed in Carnoy’s fixative for 3–4 h at 4 °C. The fixed buds were transferred into 1% acetocarmine solution in 45% acetic acid for 15–20 min at RT. On the slide, anthers were taken out of the flower buds, cut with a preparation needle in a drop of 45% acetic acid and covered with a cover glass. Then, spread preparations were prepared. After freezing in liquid nitrogen, the cover glasses were removed. Then the slides were dehydrated through a graded ethanol series and air dried. Alternatively, the slides were stored in 96% ethanol at −20 °C until use.

### 4.5. FISH Procedure

For FISH, the following probes were used:

1. pTa71 containing a 9 kb long DNA sequence of common wheat, including 18S-5.8S-26S (35S) ribosomal DNA (rDNA) [68].

2. pTa794 containing a 420 bp long DNA sequence of wheat, including 5S rDNA [69].

These DNA probes were labeled directly with SpectrumAqua and SpectrumRed fluorochromes (Abbott Molecular, Wiesbaden, Germany) respectively, by nick translation according to the manufacturer’s protocol. The FISH procedure was performed as previously described [70]. After overnight hybridization, the slides were washed in 0.1 × SSC at 50 °C for 5 min, 2 × SSC at 37 °C for 10 min, 2 × SSC at RT for 5 min and 1 × PBS at RT for 3 min. Then the slides were dehydrated through a graded ethanol series and air dried.

### 4.6. DAPI Counterstaining

After meiotic spread preparation and FISH procedures, chromosome slides were counterstained with 0.1 μg/mL DAPI (4′,6-diamidino-2-phenylindole) (Serva, Heidelberg, Germany) dissolved in Vectashield mounting medium (Vector laboratories, Peterborough, UK).

### 4.7. Analysis of Chromosomes

Chromosomal slides were examined using an Olympus BX-61 epifluorescence microscope (Olympus, Tokyo, Japan). Chromosome images were captured with a monochrome charge-coupled device camera (Cool Snap, Roper Scientific, Inc., Tucson, AZ, USA). Then, they were processed with Adobe Photoshop 10.0 (Adobe, Birmingham, AL, USA) software. At least 15 metaphase plates and 100 meiotic cells (10 plants) were analyzed for each sample. Identification of mitotic chromosomes was performed based on the morphology and distribution of revealed chromosome markers. In karyograms, chromosome pairs were set in the decreasing order of size.

### 4.8. Microanatomical Analysis of Leaves and Flowers

Epidermal peel preparation from leaves and flowers of *C. majus* samples was performed according to the standard technique [71]. The sample preparations were analyzed using an Altami BIO 2 LED biological microscope equipped with a. 3.1MP digital eyepiece USB camera (Altami, St. Petersburg, FL, USA). The images were processed with Adobe Photoshop CS3 (Adobe, Birmingham, AL, USA) software. At least 15 images for each sample of *C. majus* were examined.

## 5. Conclusions

The performed comparative agro-morphological, anatomical and molecular cytological investigation of five introduced samples of *C. majus* subsp. *majus* allowed us to estimate the intraspecific variability. At the same time, plants of the studied populations were not significantly different in most vegetative parameters or in micro-morphology of the epidermis of leaves and flowers. Moreover, chromosome aneuploidy and considerable chromosome rearrangements in their karyotypes were not detected. The analysis of chromosome behavior in meiosis also confirmed the relative genotype stability for the studied plants. Our findings indicate that plants of the newly introduced populations were adapted to the local environments: they retained a stable karyotype, possessed high yield potential, had agro-morphological differences, and therefore, they can be used in breeding programs with a view to further introduction and developing new valuable cultivars. The revealed micro-diagnostic features of leaflets and flowers, including tissue topography, anomocytic types of stomata, presence of water stomata and laticifers, peculiarities in structure of leaf mesophyll, sepals, petals and hairs, can be used for identification of *C. majus* raw materials.

## Figures and Tables

**Table 1:** Plant vegetative parameters in the studied *C. majus* populations.

Plant Vegetative Parameters	Accession Number				
	K 171-1 Control	K 211	K 176	K 117-18	K 223-14
Number of flower spikes	10.7 ± 0.7	8.0 ± 0.5	10.6 ± 0.6	9.8 ± 0.6	11.7 ± 0.9
Number of flowers per plant	121.5 ± 7.2	118.0 ± 7.0	120.5 ± 7.2	117.5 ± 6.3	125.5 ± 6.7
Flower diameter, cm	1.43 ± 0.08	1.58 ± 0.08	1.44 ± 0.08	1.37 ± 0.07	1.49 ± 0.06
Number of petals in a flower	4.0 ± 0.0	24.6 ± 1.56	4.0 ± 0.0	4.0 ± 0.0	4.0 ± 0.0
Number of seeds per fruit	50.2 ± 3.5	51.0 ± 3.6	50.1 ± 3.2	48.2 ± 3.1	51.2 ± 3.2
Length of fruits, cm	4.5 ± 0.3	4.0 ± 0.2	4.3 ± 0.2	4.2 ± 0.2	4.4 ± 0.3
Seed yield, kg/ha	18.6 ± 1.4	19.1 ± 1.7	18.2 ± 1.5	17.5 ± 1.5	19.9 ± 1.7

Values are represented as the mean value ± standard deviation.

**Table 2:** List of the studied samples of *C. majus*.

Accession Number	Origin	Developmental Characteristics
K 171-1 control	Moscow region, RF (55°33′52″ N, 37°35′30″ E)	normal-flowered
K 211	Moscow region, RF (55°33′52″ N, 37°35′30″ E)	double-flowered
K 176	Prioksko-Terrasny Nature Reserve, RF (54°54′53″ N, 37°34′19″ E)	normal-flowered
K 117-18	Petrozavodsk region, RF (61°50′57″ N, 34°19′54″ E)	normal-flowered
K 223-14	Minsk region, Belarus (54°33′57″ N, 29°09′17″ E)	normal-flowered
